# Stakeholder Perspectives on Clinical Decision Support Tools to Inform Clinical Artificial Intelligence Implementation: Protocol for a Framework Synthesis for Qualitative Evidence

**DOI:** 10.2196/33145

**Published:** 2022-04-01

**Authors:** Mohaimen Al-Zubaidy, HD Jeffry Hogg, Gregory Maniatopoulos, James Talks, Marion Dawn Teare, Pearse A Keane, Fiona R Beyer

**Affiliations:** 1 Faculty of Medical Sciences Newcastle University Newcastle upon Tyne United Kingdom; 2 The Royal Victoria Infirmary Newcastle Upon Tyne United Kingdom; 3 Faculty of Business and Law Northumbria University Newcastle Upon Tyne United Kingdom; 4 Moorfields Eye Hospital National Health Service Foundation Trust London United Kingdom; 5 Faculty of Medicine University College London London United Kingdom

**Keywords:** artificial intelligence, clinical decision support tools, digital health, implementation, qualitative evidence synthesis, stakeholders, clinical decision, decision support

## Abstract

**Background:**

Quantitative systematic reviews have identified clinical artificial intelligence (AI)-enabled tools with adequate performance for real-world implementation. To our knowledge, no published report or protocol synthesizes the full breadth of stakeholder perspectives. The absence of such a rigorous foundation perpetuates the “AI chasm,” which continues to delay patient benefit.

**Objective:**

The aim of this research is to synthesize stakeholder perspectives of computerized clinical decision support tools in any health care setting. Synthesized findings will inform future research and the implementation of AI into health care services.

**Methods:**

The search strategy will use MEDLINE (Ovid), Scopus, CINAHL (EBSCO), ACM Digital Library, and Science Citation Index (Web of Science). Following deduplication, title, abstract, and full text screening will be performed by 2 independent reviewers with a third topic expert arbitrating. The quality of included studies will be appraised to support interpretation. Best-fit framework synthesis will be performed, with line-by-line coding completed by 2 independent reviewers. Where appropriate, these findings will be assigned to 1 of 22 a priori themes defined by the Nonadoption, Abandonment, Scale-up, Spread, and Sustainability framework. New domains will be inductively generated for outlying findings. The placement of findings within themes will be reviewed iteratively by a study advisory group including patient and lay representatives.

**Results:**

Study registration was obtained from PROSPERO (CRD42021256005) in May 2021. Final searches were executed in April, and screening is ongoing at the time of writing. Full text data analysis is due to be completed in October 2021. We anticipate that the study will be submitted for open-access publication in late 2021.

**Conclusions:**

This paper describes the protocol for a qualitative evidence synthesis aiming to define barriers and facilitators to the implementation of computerized clinical decision support tools from all relevant stakeholders. The results of this study are intended to expedite the delivery of patient benefit from AI-enabled clinical tools.

**Trial Registration:**

PROSPERO CRD42021256005; https://tinyurl.com/r4x3thvp

**International Registered Report Identifier (IRRID):**

DERR1-10.2196/33145

## Introduction

### Background

Clinical artificial intelligence (AI) is a fast-growing field, demonstrating exponential increases in academic publishing, but also in the frequency of market authorizations awarded to AI-enabled computerized decision support tools (CCDSTs) [1,2]. The concept of AI has been established for more than 70 years, and varying degrees of autonomy have been designated to CCDSTs for decades [3,4]. However, it is the leap in performance brought about by the combination of rising computational capacity and neural network technology that accounts for this most recent surge in interest from various health care stakeholders [5]. Despite interest and investment from academia, industry, and policy makers, a notable paucity of real-world applications of AI-enabled CCDSTs persists [6]. This is a mark of a translational gap known as the “AI chasm” [7].

To address this AI chasm, there is a need for contemporary evidence syntheses of clinical AI research, the quantitative aspects of which have already been satisfied [8-10]. However, syntheses of health care professional (HCP) perspectives on CCDSTs of any sort are either narrow or outdated [11,12]. Meanwhile, perspectives from patients are yet to be synthesized at all. This is problematic, as the efficacy of the tools themselves are important, but tells stakeholders little about the complexity of the surrounding contextual factors that will contribute heavily to the fate of the AI chasm [13].

Clarity on implementation issues from all stakeholders is required if technological progress is to be effectively translated into patient benefit. Tailored primary qualitative research is needed to build and implement any clinical AI-enabled tool, but to optimize the design of such work, the relevant qualitative evidence base must first be robustly synthesized.

The only completed attempt to provide a synthesis of HCP perspectives specific to “AI” found only 1 study, reflecting the infancy of real-world clinical AI applications [11]. In order to synthesize a meaningful number of studies, broader search terms will be required, which reflect fewer contemporary definitions of AI, but still describe automated contributions to health care. HCP perspectives on “clinical decision support systems” were most recently synthesized from 2000-2013 publications and require updating [12]. Other planned qualitive evidence syntheses promise more contemporary findings, but have focused criteria for population, phenomena of interest, and context eligibility. These reviews will be valuable, but they fall short of what is needed to support the complex process of implementation, as they do not synthesize the breadth of relevant perspectives.

A background search of existing published syntheses, protocols, and protocol registries including PROSPERO, MEDLINE (Ovid), the Cochrane Database of Systematic Reviews, and the Joanna Briggs Institute Database of Systematic Reviews and Implementation Reports identified no duplicate protocols or reviews. Literature from partly overlapping qualitative or mixed methods syntheses were identified and fell into one or more of the three following categories: (1) a more narrow definition of the population (ie, exclusion of patient or professional perspectives or exclusion of HCPs outside of a particular discipline) [1,4,14-16]; (2) a more narrow definition of the phenomenon of interest (ie, mobile health only or AI only) [14,17,18]; and (3) a more narrow definition of the context (ie, exclusion of primary or secondary care or focus on a single specialty) [4,14,17-19].

While the overlap between these syntheses may cumulatively sample the majority of the qualitative literature relevant to the proposed review question, these multiple reviews will not generate the holistic overview that a single synthesis of all stakeholder perspectives will produce. Many are also likely to be affected by the sparsity of AI-specific literature, which has limited the efficacy of prior qualitative evidence synthesis [11]. As such, without the proposed review, a clinically important research gap will remain, limiting the efficacy with which health care policy makers and providers can work to implement clinical AI-enabled tools.

This qualitative evidence synthesis aims to consolidate the pragmatic value of primary qualitative studies of relevant stakeholders’ perspective on the implementation of CCDSTs in any health care context. In doing so, the review aims to holistically preserve the complexity of the interdependent factors that influence clinical AI implementation, supporting readers to make sense of transparent findings to address their unique implementation challenges.

### Review Question

What are the perspectives of stakeholders on using computerized clinical decision support tools and how can they inform the implementation of clinical artificial intelligence-enabled tools?

## Methods

The protocol of the proposed qualitative evidence synthesis has been registered on PROSPERO (ID 248025) and adheres to the PRISMA-P (preferred items for reporting systematic reviews and meta-analyses for protocols) 2015 checklist [20]. Best-fit framework synthesis will be conducted and reported according to ENTREQ (enhancing transparency in reporting the synthesis of qualitative research) guidelines [21,22].

### Search Strategy

An initial limited search of MEDLINE (Ovid) informed by prior quantitative and qualitative synthesis search strings was undertaken to identify articles on the topic [4,10-12]. Evidence-based search strings for the identification of qualitative literature were also used [23]. The final search used terms and synonyms around professional or lay individuals, qualitative research, CCDSTs, and health care. The text words contained in the titles and abstracts of relevant articles and the index terms used to describe the articles were used to develop a full search strategy for MEDLINE (Multimedia Appendix 1). The search strategy, including all identified keywords and index terms, was adapted for each included database. The reference list of all secondary research sources was identified and hand searched for additional studies before they themselves were excluded from synthesis. Similarly, published protocols were included at title and abstract screening to ensure published reports were captured. Where no report was identified, the corresponding authors of the protocols were contacted to ensure no report was available. Only studies indexed with an English language title and abstract were included as it is not feasible to adjust the search string for multiple languages. This is not anticipated to limit the scope of the review significantly. Studies where full text was not available in English language were translated using an automated text translation service prior to full-text screening and potential inclusion. Studies published since January 2014 were considered for inclusion, as 2014 saw the first market authorizations of clinical AI-enabled tools in Europe and America [1]. The final search was executed in April 2021, looking at studies between January 1, 2014, and April 30, 2021.

The research databases searched were MEDLINE (Ovid), Scopus, CINAHL (EBSCO), ACM Digital Library, and Science Citation Index (Web of Science). This constellation of databases was selected to support comprehensive coverage of medical, allied health professional, computer science, and grey literature, while minimizing the burden of search string translation and deduplication.

### Study Selection

**Figure 1:**
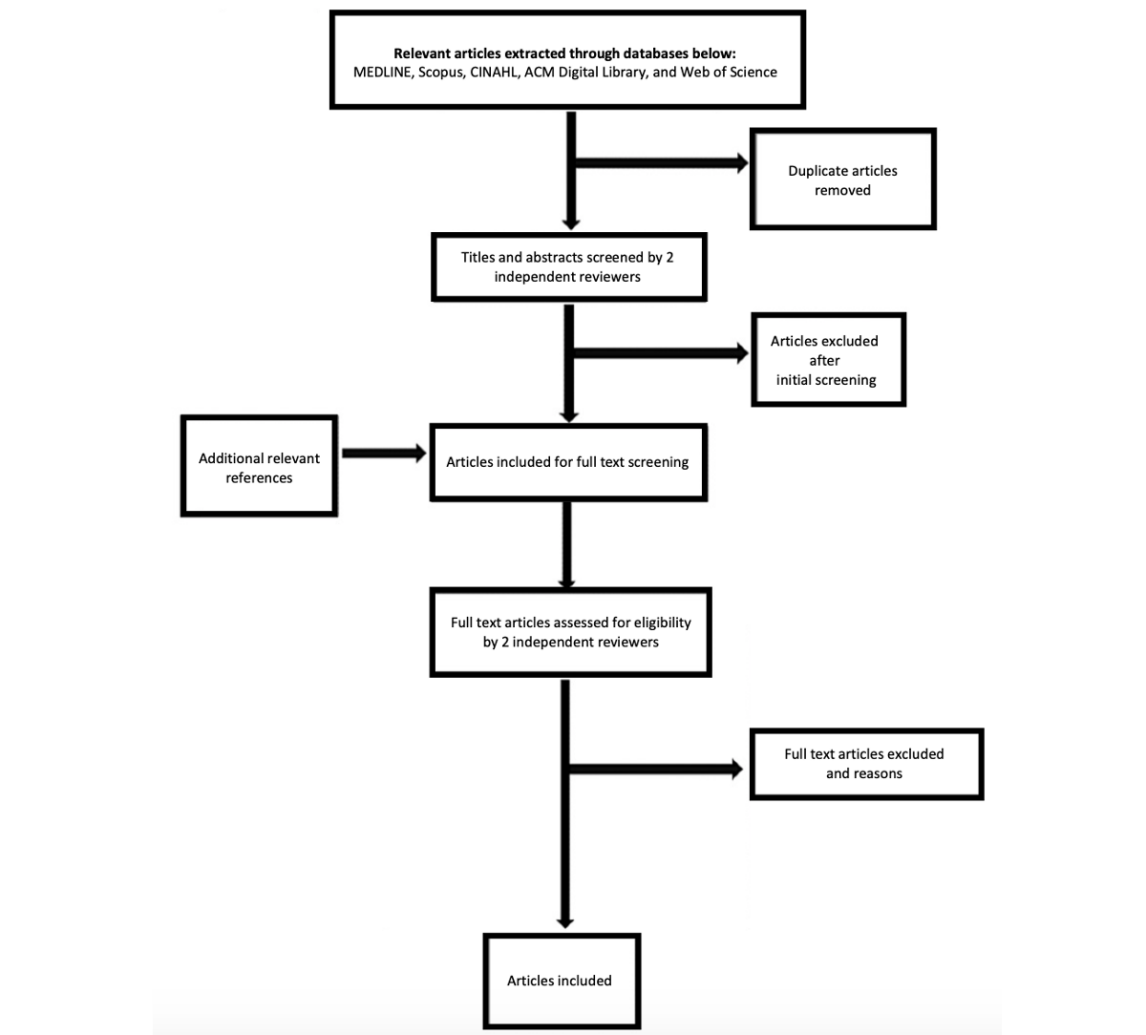
PRISMA (preferred items for reporting systematic reviews and meta-analyses)-style diagram illustrating search strategy deployment in MEDLINE (Ovid), Scopus, CINAHL (EBSCO), ACM Digital Library, and Science Citation Index (Web of Science).

Following the research database search, all identified citations were collated and uploaded into Endnote x9.3.3 (Clarivate Analytics) and deduplicated. These citations were then uploaded to Rayyan (Rayyan Systems Inc) where titles and abstracts were screened by 2 independent reviewers (JH and MA) for assessment against the review’s inclusion criteria. Potentially relevant studies were retrieved in full and assessed in detail against the inclusion criteria by 2 independent reviewers, with disagreements resolved by a third topic expert (GM). The reasons for exclusion of papers at full text that do not meet the inclusion criteria were recorded to be reported in the final report. Eligible full texts will be imported into NVivo Release 1.2.426 (QSR International) for coding. The results of the search and the study inclusion process will be reported in full in the final report and presented in a PRISMA flow diagram (Figure 1) [24].

### Inclusion Criteria

#### Participants

The participants included are patients, caregivers, or HCPs using or accessing fully or partly automated CCDSTs.

#### Phenomena of Interest

Of interest to this study are the stakeholders’ perspectives of fully or partly automated CCDST implementation in a real-world or hypothetical setting.

#### Context

The context of this study will be all real-world, clinical trial, or hypothetical health care settings worldwide published during or after January 2014.

#### Types of Studies

This review will consider primary studies that focus on textual qualitative data, including but not limited to designs such as phenomenology, grounded theory, ethnography, action research, and feminist research.

### Exclusion Criteria

#### Phenomena of Interest

Articles that focus exclusively on computerized treatments, physical tools, information sharing, data storage, or data collection methods were not eligible. Such phenomena included computerized cognitive behavioral therapy, telemedicine applications, noninteractive decision aids, robot companions, nonautonomous robotic surgical instruments, electronic health records, and data collection smartphone apps. However, if articles consider these phenomena alongside a fully or partly automated CCDST and meet the other inclusion criteria, they were deemed eligible.

### Assessment of Methodological Quality

Eligible primary studies will be critically appraised by 2 independent reviewers for methodological quality using the standard Joanna Briggs Institute Critical Appraisal Checklist for Qualitative Research [25]. The authors of the papers will be contacted to request missing or additional data for clarification, where required. Any disagreements that arise between the reviewers will be resolved through discussion, or with a third reviewer arbitrating if necessary. The results of critical appraisal will be reported in narrative form and in a table.

All included items, regardless of the results of their methodological quality, will undergo data extraction and synthesis, where possible. The results of critical appraisal will be used to describe the credibility of findings and to help interpret contradictions between the included studies.

### Data Extraction

Data will be extracted from the studies included in the review by 2 independent reviewers (JH and MA) using NVivo Release 1.2.426. Data extraction will include publication date, study methods, health care context, population size and characteristics, phenomenon of interest, geographical location, and quality of each study. The findings and their illustrations, which relate to this review’s phenomena of interest, will be extracted and assigned a level of credibility based on the strength of support offered by illustrations. A proportion of data collection will be performed in parallel by both reviewers in order to develop a consistent approach at the outset and to check that agreement is maintained throughout the process. Any disagreements that arise between the reviewers will be resolved through discussion, or with input from topic (GM) and method (FB) experts where disagreements persist. The authors of the papers will be contacted to request missing or additional data, where required.

### Data Synthesis

**Figure 2:**
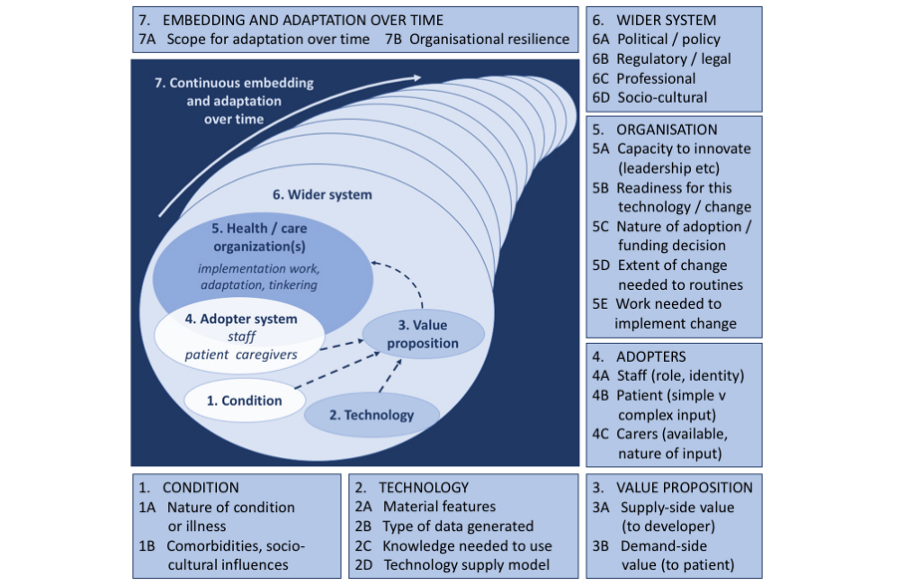
The Nonadoption, Abandonment, Spread, Scale-up and Sustainability (NASSS) model reproduced from the original open access publication.

A total of 22 a priori themes established by the Nonadoption, Abandonment, and Challenges to the Scale-Up, Spread, and Sustainability (NASSS) framework will be used for this best-fit framework synthesis (Figure 2). The NASSS framework was considered as an appropriate framework as it outlines the interacting complexity of factors and related stakeholders at the policy, organizational, and practice level, shaping the implementation of digital innovations [13,26]. Two reviewers will carry out “line-by-line” coding in NVivo, to identify the findings from the included studies while associating them with the contributing study’s descriptive data. Where these findings do not translate into the preexisting themes of NASSS, an inductive approach will be used by the reviewer to create an additional theme as per best-fit framework synthesis methodology [22]. Assigning findings to a priori themes and creating new themes will be performed by individual reviewers in the first instance. These decisions will then be critically reviewed through a series of meetings with all authors and 6 UK-based lay representatives with a range of health perspectives.

### Assessing Confidence in the Findings

The transparent reporting of the critical appraisal method will support appraisal of the findings. A subjective assessment of the relevance of the synthesized findings to the population of patients, caregivers, and health care professionals who it ultimately intends to serve will take place through discussion with health service stakeholders in a related subsequent primary qualitative research study.

### Statistical Methods

Data concerning the final articles’ characteristics including a quality score, the year and type of publication, source title and field, source impact factor, implementation context, theoretical approach use, study methods, and study participants will also be collected. These data will undergo the Kendell rank correlation coefficient testing and logistical regression analysis to identify trends and associations within eligible studies’ characteristics.

### Ethics Approval

This study has been granted ethical approval from the Health Research Authority (IRAS:280448).

## Results

Study registration was obtained from PROSPERO (ID 248025) in May 2021. The search string was executed across the 5 databases in April 2021, yielding 4437 potentially eligible articles after initial deduplication. Abstract and full text screening is ongoing and due to be completed in late August 2021, with data extraction and quality appraisal set to commence following this stage. We expect to have completed full text data extraction by October 2021, with results expecting to be published in late 2021. We aim to publish the manuscript with open access in a peer-reviewed journal with conference presentations targeted to HCP, policy makers, and industry stakeholder groups. While the NASSS framework offers a powerful sense-making tool to analyze the breadth of data, it may not be immediately accessible to those without a background in social sciences. Consequently, the analysis will be centered on NASS, but the presentation of results will be categorized by the spheres of influence of stakeholder groups that arise from the data.

## Discussion

### Context

While publication regarding the efficacy of AI-enabled clinical tools has surged in the past 20 years, our pilot searches suggest a much more modest volume of literature exploring views of key stakeholders [2]. The qualitative research methods used in this literature offer a more sensitive tool to understand and manage the complexity surrounding CCDST implementation [27]. The proposed work aims to distil the practical value of the existing evidence base in order to make its value more accessible to implementation academics and practitioners working with AI. It will identify gaps in our understanding and help to inform meaningful future work, informing health care policy and future implementation of CCDSTs. Specifically, it will provide analysis of the potential barriers and facilitators to the implementation of AI-enabled CCDSTs across all health care settings, making a contribution with broad relevance. This is important given the strategic emphasis placed on AI-enabled clinical tools by health policy makers worldwide [28,29]. To the best of our knowledge, this is the first planned study that will synthesize the perspective of patients, HCPs, academics, and policy makers on CCDSTs.

### Strengths and Limitations

One of the limitations of this review is finding the balance between accuracy through microlevel analysis and pragmatism from a macrolevel. We have attempted to minimize this by adopting the NASSS framework, which facilitates multilevel analysis and a full range of stakeholder perspectives, accepting that there will be some compromise in targeting this pragmatic goal. This is an intentional limitation, as it is only through considering the topic as a whole that we are able to meaningfully examine the complexity of implementation.

The proposed synthesis does not examine health, economic, or other quantitative data concerning CCDST. While such analyses are crucial to implementation, they are outside the scope of this protocol, which already covers an ambitious breadth of data. However, qualitative reflections of stakeholders’ perceptions of cost and value that arise in the data will be analyzed.

Another limitation is the varied definitions of clinical AI used within the literature. We have taken a pragmatic approach in considering that the focus of clinical AI involves the partial or full surrender of autonomy from the practitioner. Consequently, we constructed eligibility criteria that included a priori rules based on CCDST, as well as “black box” CCDST, such as those using convolutional neural networks. Consequently, some of the perspectives raised may not relate directly to the implementation of contemporary definitions of clinical AI, but we will be transparent in reporting the characteristics of eligible articles in order to support readers’ accurate interpretation.

### Conclusions

This paper describes the protocol for a qualitative evidence synthesis aiming to consolidate the perspectives of stakeholders from all health care contexts on CCDST implementation. We hope the results of this study will influence the design of future research and health care policy to expedite patient benefit from AI-enabled clinical tools.

## Appendix

Multimedia Appendix 1 Search strategy.

## Abbreviations

AI: artificial intelligence
CCDST: computerized clinical decision support tool
ENTREQ: enhancing transparency in reporting the synthesis of qualitative research
HCP: health care professional
NASSS: Nonadoption, Abandonment, and Challenges to the Scale-Up, Spread, and Sustainability
PRISMA-P: preferred items for reporting systematic reviews and meta-analyses for protocols
