# Supplementary material for: Stakeholder Perspectives on Clinical Decision Support Tools to Inform Clinical Artificial Intelligence Implementation: Protocol for a Framework Synthesis for Qualitative Evidence
Source: JMIR Res Protoc. 2022 Apr 1;11(4):e33145. doi: 10.2196/33145 (PMC9015736; doi:10.2196/33145)
Supplement: Multimedia Appendix 1 [file resprot_v11i4e33145_app1.docx]

## Multimedia Appendix

## S1 – Search Strategy

Ovid MEDLINE(R) <1996 to April Week 4 2021>

1 (ethnological research or ethnograph* or life stor* or women* stor* or social construct* or postmodern* or post-structural* or post structural* or poststructural* or post modern* or post-modern* or feminis* or interpretative or interpretive action research or cooperative inquir* or co operative inquir* or co-operative inquir* or existential or unstructured or openended or open ended or life world or life-world or conversation analys?s or personal experience* or theoretical saturation or cluster sampl* or glaser* or participant observ* or human science or biographical method or heidegger* or colaizzi* or spiegelberg* or husserl* or foucault* or mixed method* or mixed-method*).ab,kf,kw,ti. 77395

2 (corbin* adj2 strauss*).ab,kf,kw,ti. 252

3 (field adj (study or studies or research)).ab,kf,kw,ti. 11952

4 (data adj1 saturat*).ab,kf,kw,ti. 851

5 ((discourse* or discurs*) adj1 analys?s).ab,kf,kw,ti. 1699

6 (van adj (manen* or kaam*)).ab,kf,kw,ti. 405

7 (merleau adj ponty*).ab,kf,kw,ti. 178

8 ((interpretative or interpretive) adj (approach or research or data or method* or paradigm)).ab,kf,kw,ti. 609

9 (experiential adj (qualitative or knowledge or method*)).ab,kf,kw,ti. 416

10 ((lived or life) adj experience*).ab,kf,kw,ti. 9388

11 ((theme* or thematic) adj1 (analys?s or data or synthesis or research)).ab,kf,kw,ti. 18530

12 (account* adj1 (participant or patient* or clinician* or user* or professional* or carer* or family or stakeholder or open-ended or unstructured)).ab,kf,kw,ti. 2953

13 (ethnonursing or phenomenol* or theoretical sampl* or observational method* or content analysis or emic or etic or hermeneutic* or semiotic*).af. 45073

14 (narrative* adj (analys?s or synthes?s or data or research or methods or inquiry)).af. 5249

15 (constant adj (comparative or comparison)).af. 4045

16 (grounded adj (theor* or study or studies or research or analys?s)).af. 10396

17 (purpos* adj sampl*).af. 8771

18 (focus adj group*).af. 45407

19 (qualitative adj1 (research or method* or data or study or studies or paradig* or analy*)).af. 110442

20 Qualitative Research/ or Interview/ or Nursing Methodology Research/ or exp Diffusion of Innovation/ 109507

21 (Artificial intelligence or Boltzmann machine* or Long short-term memory or Gated recurrent unit or Rectified linear unit or Autoencoder or Backpropagation or Multilayer perceptron or Convnet or Support vector machine or Random forest or Lasso or Kernel or Elastic net* or Bayesian or Naive bayes or Genetic algorithm).ab,kf,kw,ti. 78267

22 ((deep or convolutional or bayesian or neural or elastic) adj1 net*).ab,kf,kw,ti. 40303

23 ((machine or deep or reinforcement or ensemble or convolutional) adj1 learning).ab,kf,kw,ti. 32394

24 Big data/ or Decision support system, clinical/ or exp Algorithms/ 329733

25 ((algorithm* or computeri* or computer-based or computer based or machine-based or machine based or Computer assisted or Computer-assisted or Computer aided or Computer-aided or integrat* or technolog* or digital or electron*) adj3 (decision support or decision-support or decision aid or decision-aid)).ab,kf,kw,ti. 2325

26 exp Health Occupations/ or exp Health Personnel/ or exp Persons/ 7265934

27 (Perspective* adj1 (patient* or carer* or clinician* or doctor* or stakeholder* or nurse*)).ab,kf,kw,ti. 11028

28 1 or 2 or 3 or 4 or 5 or 6 or 7 or 8 or 9 or 10 or 11 or 12 or 13 or 14 or 15 or 16 or 17 or 18 or 19 or 20 or 27 298429

29 21 or 22 or 23 or 24 or 25 396074

30 26 and 28 and 29 1856

31 limit 30 to (humans and yr="2014 -Current") 1093
